
JOURNAL INFORMATION
==============================
NLM Title Abbreviation: Hum Brain Mapp
Journal ID: Hum Brain Mapp
NLM Journal ID: 9419065
Journal ID: HBM

Human Brain Mapping

ISSN: 1065-9471
EISSN: 1097-0193
Publisher: Wiley

ARTICLE INFORMATION
==============================
PMCID: PMC11474524
DOI: 10.1002/hbm.25934
Article ID: HBM25934
Article version: 1
Subjects: Issue Information, Issue Information

Editorial board ‐ TOC

Electronic publication date: 2023 Sep 1
Publication date: 2023 Oct 1
Volume: 44
Issue: 14
First page: 4831
Last page: 4832
Copyright: © 2023 The Authors. Human Brain Mapping published by Wiley Periodicals LLC.
License: This is an open access article under the terms of the http://creativecommons.org/licenses/by/4.0/ License, which permits use, distribution and reproduction in any medium, provided the original work is properly cited.
License URL: https://creativecommons.org/licenses/by/4.0/

Figure count: 0
Table count: 0
Page count: 7

source-schema-version-number: 2.0
cover-date: 1 October, 2023
details-of-publishers-convertor: Converter:WILEY_ML3GV2_TO_JATSPMC version:6.4.9 mode:remove_FC converted:15.10.2024

==============================

### HUMAN BRAIN MAPPING

Open Access

Volume 44, Issue 14, 1 October, 2023

View this journal online at wileyonlinelibrary.com

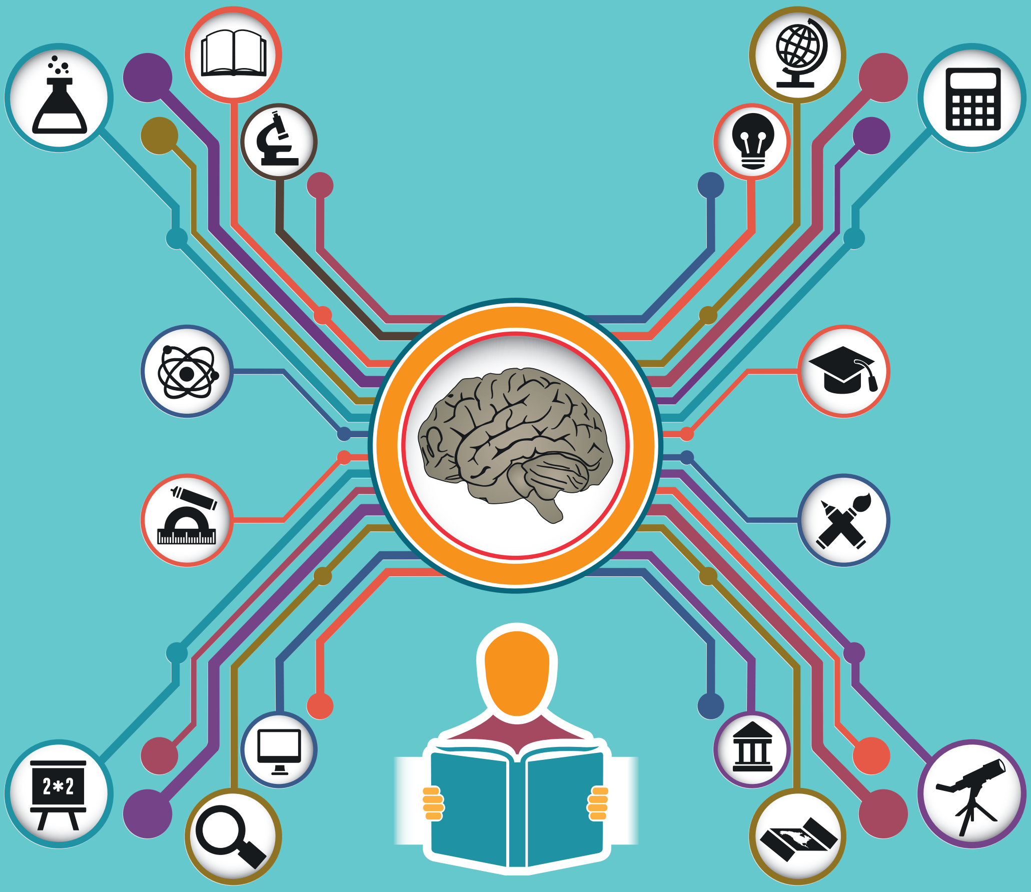

WILEY

ISSN 1097-0193

### HUMAN BRAIN MAPPING

### A Journal Devoted to Functional Neuroanatomy and Neuroimaging

### *Editor-in-Chief*

**Simon B. Eickhoff**
Institute for Systems Neuroscience
Heinrich-Heine University, Düsseldorf, Germany

### *Deputy Editors-in-Chief*

**Gary Egan**
Monash Biomedical Imaging
Monash University, Victoria, Australia

**Sophia Frangou**
Icahn School of Medicine
at Mount Sinai, New York, USA

**Martijn van den Heuvel**
Brain Center Rudolf Magnus,
University of Medical Center,
Utrecht, Netherlands

### *Associate Editors*

**Annabel Chen**
Nanyang Technological University, Singapore

**Michael J. Farrell**
Monash University, Victoria, Australia

**Massimo Filippi**
Università Vita-Salute San Raffaele, Milan,
Italy

**Sophia Frangou**
Icahn School of Medicine at Mount Sinai
New York, USA

**Cheryl L. Grady**
Rotman Research Institute
Toronto, Canada

**Joachim Gross**
Centre for Cognitive Neuroimaging
University of Glasgow, Glasgow, UK

**Martin A. Lindquist**
John Hopkins, Bloomberg School of Public
Health, MD, USA

**Tomáš Paus**
Rotman Research Institute
Toronto, Canada

**Indira Tendolkar**
Radboud University, Nijmegen,
The Netherlands

**Paul Thompson**
Laboratory of Neuro Imaging,
University of Southern California,
Los Angeles, CA, USA

**Helen Zhou**
Yong Loo Lin School of Medicine, National
University of Singapore, Singapore

### *Editorial Board*

**Susan Bookheimer**
Center for Cognitive Neuroscience
UCLA School of Medicine, USA

**Allen R. Braun**
NIDCD, National Institutes of Health
Bethesda, USA

**Christian Buechel**
Univ-Krankenhaus Eppendorf
Hamburg, Germany

**Marco Catani**
King's College London, UK

**Franco Cauda**
University of Turin, Italy

**Vincent Clark**
University of New Mexico, USA

**Olivier David**
Université Joseph Fourier, France

**Mark D'Esposito**
University of California, Berkeley,
USA

**Julien Doyon**
Functional Neuroimaging Unit
University of Montreal, Canada

**Eugene Duff**
University of Oxford, UK

**Timothy Q. Duong**
Research Imaging Institute,
University of Texas Health Science
Center, USA

**Paul Fletcher**
University of Cambridge, UK

**Jia-Hong Gao**
Brain Research Imaging
Center The University of Chicago,
USA

**David C. Glahn**
Yale University School of Medicine
Hartford, USA

**Gregory S. Hickok**
Center for Cognitive
Neuroscience University of California,
Irvine, USA

**Barry Horwitz**
NIDCD, National Institutes of Health
USA

**Peter Jezzard**
John Radcliffe Hospital
University of Oxford, UK

**David N. Kennedy**
Harvard Medical School, USA

**Neda Ladbon-Bernasconi**
Montreal Neurological Institute
Canada

**Nicholas Lange**
Harvard Medical School, USA

**Tatia M. C. Lee**
State Key Laboratory of Brain &
Cognitive Sciences, The University of
Hong Kong

**Louis Lemieux**
UCL Institute of Neurology, UK

**Bernard Mazoyer**
CNRS/CEA, Université de Bordeaux
France

**Gregory McCarthy**
Yale University, USA

**A. Randy McIntosh**
Rotman Research Institute
Toronto, Canada

**Janine Mendola**
McGill Vision Research
McGill University, Canada

**Thomas Nichols**
University of Warwick, UK

**William Penny**
Wellcome Trust Centre for
Neuroimaging, UCL Institute of
Neurology, UK

**Laurent Petit**
CNRS, Université de Bordeaux,
France

**Russel A. Poldrack**
Stanford Unversity, USA

**Jean-Baptiste Poline**
Imagen Orsay Service Hospitalier
Frédéric Joliot, France

**Stephen Smith**
Center for Functional MRI of the
Brain, University of Oxford, UK

**Elizabeth Sowell**
Children'sHospital Los Angeles, USA

**Li Hai Tan**
Shenzhen Institute of Neuroscience
Shenzhen, P.R. China

**Kate Watkins**
John Radcliffe Hospital
University of Oxford, UK

**Richard G. Wise**
Cardiff University, UK

**Roger Woods**
UCLA School of Medicine, USA

**Mark Woolrich**
Oxford Centre for Human Brain
Activity, University of Oxford, UK

**Jinhu Xiong**
University of Iowa, USA

**Robert Zatorre**
MontrealNeurological Institute, Canada

**Open Access and Copyright**
All articles published by *Human Brain Mapping* submitted after January 1, 2020 are fully open access: immediately freely available to read, download, and share. All articles accepted from August 6, 2019 are published under the terms of a Creative Commons license.
Copyright on any research article published by *Human Brain Mapping* is retained by the author(s).

Further information about open access licenses and copyright can be found at: https://authorservices.wiley.com/open-research/open-access/index.html. Information on the licenses used by *Human Brain Mapping* can be found at: https://onlinelibrary.wiley.com/journal/10970193.

**Disclaimer**
The Publisher and Editors cannot be held responsible for errors or any consequences arising from the use of information contained in this journal; the views and opinions expressed do not necessarily reflect those of the Publisher and Editors, neither does the publication of advertisements constitute any endorsement by the Publisher and Editors of the products advertised. Wiley Open Access articles posted to repositories or websites are without warranty from Wiley of any kind, either express or implied, including, but not limited to, warranties of merchantability, fitness for a particular purpose, or non-infringement. To the fullest extent permitted by law Wiley disclaims all liability for any loss or damage arising out of, or in connection, with the use of or inability to use the content.

### HUMAN BRAIN MAPPING

A Journal Devoted to
Functional Neuroanatomy
and Neuroimaging

VOLUME 44, NUMBER 14, 1 OCTOBER 2023

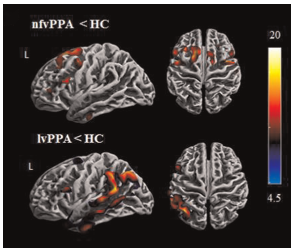

*See article by, Hinkley et al, pages 4833-4847, this issue*

### CONTENTS

◆ *4833* Distinct neurophysiology during nonword repetition in logopenic and non-fluent variants of primary progressive aphasia
**Leighton B. N. Hinkley, Megan Thompson, Zachary A. Miller, Valentina Borghesani, Danielle Mizuiri, Wendy Shwe, Abigail Licata, Seigo Ninomiya, Michael Lauricella, Maria Luisa Mandelli, Bruce L. Miller, John Houde, Maria Luisa Gorno-Tempini, and Srikantan S. Nagarajan**
***Published online 30 July 2023***

◆ *4848* Sex-related patterns in the electroencephalogram and their relevance in machine learning classifiers
**Thomas Jochmann, Marc S. Seibel, Elisabeth Jochmann, Sheraz Khan, Matti S. Hämäläinen, and Jens Haueisen**
***Published online 17 July 2023***

◆ *4859* Single-subject electroencephalography measurement of interhemispheric transfer time for the in-vivo estimation of axonal morphology
**Rita Oliveira, Marzia De Lucia, and Antoine Lutti**
***Published online 20 July 2023***

◆ *4875* Style transfer generative adversarial networks to harmonize multisite MRI to a single reference image to avoid overcorrection
**Mengting Liu, Alyssa H. Zhu, Piyush Maiti, Sophia I. Thomopoulos, Shruti Gadewar, Yaqiong Chai, Hosung Kim, and Neda Jahanshad, for the Alzheimer's Disease Neuroimaging Initiative**
***Published online 20 July 2023***

(*continued on overleaf*)

(*continued from overleaf*)

◆ *4893* BIANCA-MS: An optimized tool for automated multiple sclerosis lesion segmentation
**Giordano Gentile, Mark Jenkinson, Ludovica Griffanti, Ludovico Luchetti, Matteo Leoncini, Maira Inderyas, Marzia Mortilla, Rosa Cortese, Nicola De Stefano, and Marco Battaglini**
***Published online 2 August 2023***

◆ *4914* Bloody noise: The impact of blood-flow artifacts on registration
**John D. Lewis, Vladimir S. Fonov, and D. Louis Collins**
***Published online 29 July 2023***

◆ *4927* Enhanced functional connectivity in the reward circuitry in healthy adults with weekend catch-up sleep
**Keun-Tae Kim, Hayom Kim, Jooheon Kong, and Jung Bin Kim**
***Published online 19 July 2023***

◆ *4938* Modeling venous bias in resting state functional MRI metrics
**Julia Huck, Anna-Thekla Jäger, Uta Schneider, Sophia Grahl, Audrey P. Fan, Christine Tardif, Arno Villringer, Pierre-Louis Bazin, Christopher J. Steele, and Claudine J. Gauthier**
***Published online 27 July 2023***

◆ *4956* Reliability and validity of brain-gastric phase synchronization
**Gidon Levakov, Shira Ganor, and Galia Avidan**
***Published online 1 August 2023***

◆ *4967* Correction to “Deep multimodal predictome for studying mental disorders”
***Published online 29 July 2023***
